# A Rare Bacterium Isolated in the Blood Cultures of an ICU Patient Monitored for Type 2 Respiratory Failure: Myroides odoratimimus

**DOI:** 10.7759/cureus.102765

**Published:** 2026-02-01

**Authors:** Kamil Gönderen, Birhan Çetin, Tuba Müderris

**Affiliations:** 1 Internal Medicine, İzmir Katip Çelebi University Faculty of Medicine, İzmir, TUR; 2 Medical Microbiology, İzmir Katip Çelebi University Faculty of Medicine, İzmir, TUR

**Keywords:** blood stream infections, hypercapneic respiratory failure, intensive and critica care, myroides odoratimimus, opportunitic infection

## Abstract

*Myroides* species are rare opportunistic pathogens that are not part of the normal human flora and can cause infections, particularly in immunocompromised individuals. They are aerobic, non-motile, yellow-pigmented, oxidase-positive, gram-negative, rod-shaped bacteria commonly found in environmental sources such as water and soil. In this case presentation, *Myroides odoratimimus* is reported as the causative agent of a bloodstream infection in a patient with a prolonged ICU stay due to chronic obstructive pulmonary disease (COPD), type 2 respiratory failure, and acute renal failure.

## Introduction

The *Myroides* species were initially designated *Bacterium faecale aromaticum* and *Flavobacterium odoratum*. In 1996, the species were reclassified as *Myroides odoratus* and *Myroides odoratimimus*, following the application of DNA-rRNA hybridization methods [1]. These organisms are characterised as aerobic, non-motile, yellow-pigmented, oxidase-positive, gram-negative rods, and their natural habitats are understood to be water and soil. *Myroides* species have been observed to exhibit multidrug resistance and are not part of the normal human flora. It is noteworthy that such infections rarely occur in patients with compromised immune systems. As opportunistic pathogens, they have the capacity to cause life-threatening infections in patients with underlying diseases.

*Myroides* species are difficult to treat due to their increased antibiotic resistance. Studies investigating antibiotic resistance have not yielded clear information about resistance genes, resistance islands, or enzymes. This case report presents a case of *M. odoratimimus* isolated from a blood culture of a patient with chronic obstructive pulmonary disease (COPD) and acute renal failure who had been in the ICU for a protracted period.

## Case presentation

A 79-year-old male presented to an external emergency service in March 2025 with complaints of cough and sputum. He was found to be tachypneic and somnolent and was admitted to the ICU with a pre-diagnosis of type 2 respiratory failure. On the third day, due to worsening respiratory failure, the patient was electively intubated. His medical history included COPD for 15 years, treated with inhaled corticosteroids, and coronary artery disease with prior stent placement ten years ago. Due to repeated extubation and reintubation episodes, he was transferred to the ICU of Atatürk Training and Research Hospital, Izmir Katip Celebi University (İzmir, TUR), in May 2025 for further evaluation and extubation. On admission, his body temperature was 37.1°C, heart rate 130/bpm, blood pressure 90/60 mmHg (on norepinephrine 25 mcg/min), oxygen saturation 93% on mechanical ventilation in adaptive pressure ventilation with synchronized intermittent mandatory ventilation (APV SIMV) mode (tidal volume: 460 mL (7 mL/kg), respiratory rate: 18/min, positive end-expiratory pressure (PEEP): 5 cmH₂O). The patient's laboratory values ​​at the time of septic shock are shown in Table 1.

**Table 1:** Laboratory values at the time of the patient's septic shock

Parameter	Value
Leukocyte	55.26x10^9^
Neutrophil	48.1x10^9^(87%)
C-reactive protein	136.5 mg/L
Procalcitonin	1.85 ug/L
Blood urea nitrogen	62 mg/dl
Creatinine	2.99 mg/dl

Suspected septic shock led to the removal of two femoral catheters. Samples were obtained for analysis, including blood, urine, tracheal aspirate, and pressure ulcer cultures. The patient, who had a history of receiving ceftazidime-avibactam and tigecycline treatment for 14 days due to *Klebsiella pneumoniae* growth in tracheal aspirate culture obtained at an outside facility, was continued on polymyxin B (500,000 IU × 3.75 loading dose followed by 2 × 2.25 maintenance dose every 12 hours), meropenem (1 g twice daily via three-hour infusion), and daptomycin (350 mg every 48 hours) therapy initiated two days earlier at the outside center due to persistent septic shock. Due to prolonged intubation, a tracheostomy was performed, and follow-up continued. As *Candida auris* growth was detected in a blood culture obtained during hospitalization, anidulafungin (200 mg loading dose followed by a 100 mg daily maintenance dose) was added to the treatment.

In addition, pan-resistant *Acinetobacter baumannii* and *K. pneumoniae* (resistant to amoxicillin, levofloxacin, amikacin, piperacillin/tazobactam, ceftazidime/avibactam, carbapenems, and trimethoprim/sulfamethoxazole) were detected in the blood culture. Tracheal aspirate culture revealed growth of *Pseudomonas aeruginosa* (10⁴ CFU/mL), A. baumannii (10⁴ CFU/mL), and *Achromobacter xylosoxidans* (10⁴ CFU/mL). However, the tracheal aspirate was not considered significant because the leukocyte count was <10 per field. *Pseudomonas aeruginosa* (10⁵ CFU/mL) was isolated from the urine culture. Following the detection of *C. auris* in the blood culture, subsequent blood cultures taken every other day showed growth of *P. aeruginosa* and *K. pneumoniae*. The isolates were susceptible only to ceftazidime/avibactam. As a result, ceftazidime/avibactam (0.8/0.2 g twice daily) was added to the treatment regimen.

Due to persistent *C. auris* growth in follow-up blood cultures despite anidulafungin therapy, treatment was switched to micafungin (100 mg/day). Ophthalmological consultation ruled out ocular involvement. Transthoracic echocardiography was performed, and no valvular vegetations were observed. On day 15 of hospitalization, due to increased vasopressor and inotropic support requirements, blood, urine, and tracheal aspirate cultures were obtained, and amikacin (550 mg/day) was added to the treatment.

**Figure 1:**
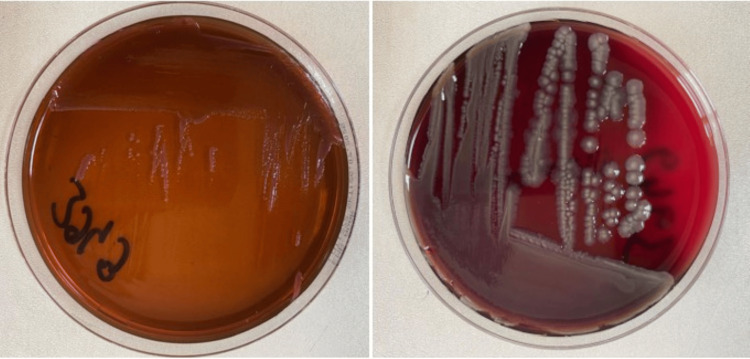
M. odoratimimus colonies on EMB agar (left) and 5% sheep blood agar (right) EMB: Eosin-methylene blue

As *C. auris* growth persisted in blood cultures taken every other day despite micafungin therapy, amphotericin B (225 mg/day) was initiated. Due to clinical deterioration, two consecutive sets of blood cultures were obtained from both arms and incubated in the automated BACTEC FX (Becton Dickinson, Franklin Lakes, NJ, USA) system. After a positive signal, Gram staining revealed Gram-negative bacilli. Subcultures were performed from the blood culture bottles onto eosin-methylene blue (EMB) agar, 5% sheep blood agar, and chocolate agar, and incubated aerobically at 37°C for 24 hours. Following incubation, all three media showed round, mucoid, yellow-pigmented colonies with a fruity odor. Gram staining of the colonies revealed Gram-negative bacilli. The isolates were identified as *M. odoratimimus* using matrix-assisted laser desorption/ionization time-of-flight mass spectrometry (MALDI-TOF MS; Bruker Scientific Instruments, Billerica, MA, USA) (Figure 1). Antimicrobial susceptibility testing was performed both by the Phoenix automated system (Becton Dickinson) and the standard disk diffusion method (Table 2). 

**Table 2:** Antibiotic susceptibility test of M. odoratimimus

Antimicrobial susceptibility testing	Minimum inhibitory concentration results from the automated system	Antibiotics tested via standard disk diffusion
Amikacin	>32	30 µg
Ceftazidime	>8	30 µg
Cefepime	>8	
Imipenem	>8	10 µg
Piperacilin/tazobactam	>16/4	30/6 µg
Ciprofloxacin	>4	5 µg
Levofloxacin	>2	5 µg
Colistin	>4	
Gentamicin		10 µg
Tobramycin		10 µg
Cefazolin		30 µg
Cefixime		50 µg
Ceftriaxone		30 µg
Ertapenem		10 µg
Ampicilin		2 µg
Amoxicillin/clavulanate		20/10 µg
Trimethoprim/sulfamethoxazole		25 µg
Fosfomycin		50 µg
Nitrofurantoin		300 µg
Tigecycline		15 µg

Cultures taken after the patient went into septic shock revealed the presence of *Myroides*. No growth was detected in other cultures. The patient's *Myroides* growth was considered the primary pathogen, and the antibiotic treatment was revised accordingly. The patient, whose need for inotropic support progressively increased, died of septic shock three days after the isolation of pan-resistant *M. odoratimimus*.

## Discussion

Although *Myroides *species are not considered part of the normal human flora and are instead detected in cultures of immunocompromised patients, they were hypothesized to be the causative agent in the present patient. This patient was not immunocompromised but had a prolonged stay in the ICU. Infections caused by the *Myroides* species are mostly observed in immunocompromised patients, those with prolonged hospital stays, and individuals with indwelling medical devices. In the literature, there are reported cases of urinary tract infections, necrotizing fasciitis, soft tissue infections, pneumonia, and sepsis caused by *Myroides* species in immunocompromised individuals. The first outbreaks of Myroides species were reported in Tunisia and Romania. In these outbreaks, invasive procedures such as kidney transplantation, radical cystectomy, and transurethral resection were identified as risk factors [2-4]. Urinary catheters are frequently used in ICUs, and most case reports related to Myroides species in the literature are associated with urinary catheterization. These reports emphasize the therapeutic challenge due to multidrug resistance [5].

The ability of *Myroides* species to form biofilms and their multidrug resistance contribute significantly to the difficulty in treating infections caused by these microorganisms [6]. Their production of beta-lactamase enzymes leads to resistance against most beta-lactam antibiotics, including carbapenems. Another important factor contributing to antimicrobial resistance is the potential for horizontal transfer of resistance genes via plasmids among *Myroides* species [7,8]. It is also possible for *Myroides *species to cause infections in immunocompetent patients. The absence of immunosuppression may improve survival outcomes. Lu et al. reported a case of catheter-related bloodstream infection caused by* M. odoratimimus* in an immunocompetent patient [9]. The isolate was susceptible to cefoperazone/sulbactam, and the patient was discharged following appropriate antibiotic therapy.

Kurt et al. reported a case of pan-resistant *Myroides* isolated from a patient in the ICU with severe COVID-19 pneumonia. The patient required renal replacement therapy due to acute renal failure and extracorporeal membrane oxygenation (ECMO) due to hypercapnia/hypoxia. Despite antibiotic therapy, the immunosuppressed patient with a central venous catheter could not be saved [10].

Kadri et al. described the development of *M. odoratimimus* infection in a patient who underwent external ventricular drainage due to hemorrhagic stroke. The patient was treated with meropenem and vancomycin [11]. The use of medical devices and the immunosuppressed status of the patient were consistent with other reported cases.

Kutlu et al. reported the isolation of *M. odoratimimus* strains from the urine samples of six ICU patients over a three-month period. All patients were immunocompromised and had urinary catheters, and all isolates were pan-resistant [12]. Licker et al. reported the isolation of *M. odoratimimus* in four patients hospitalized in urology, diabetes, and surgical units [13]. Lovering et al. documented a case of *Myroides* infection resulting from crab shell injury in a 76-year-old patient with non-alcoholic steatohepatitis-related cirrhosis. The pathogen was susceptible to levofloxacin, and the patient was discharged following a 14-day course of levofloxacin 500 mg/day [14].

The selection of an appropriate antimicrobial therapy for infections can be challenging due to the highly resistant nature of these bacteria to most antibacterial agents and the limited clinical experience. With increased awareness of *Myroides* infections, which are highly antibiotic-resistant, adherence to infection control measures aimed at preventing cross-contamination, patient isolation, removal of invasive catheters, identification of patient risk factors, and investigation of environmental factors is extremely important.

## Conclusions

The use of invasive devices is common in ICUs. In immunosuppressed patients, such as those with malignancy, diabetes mellitus, or a history of steroid use, *Myroides* species should be considered as potential infectious agents. Additionally, it should be kept in mind that these rarely encountered *Myroides* species may cause outbreaks through cross-contamination. Adherence to infection control measures aimed at preventing cross-transmission of these highly antibiotic-resistant organisms is critically important.
